# Pharmacophore Modeling and Binding Affinity of Secondary Metabolites from *Angelica keiskei* to HMG Co-A Reductase

**DOI:** 10.3390/molecules29132983

**Published:** 2024-06-23

**Authors:** Diah Lia Aulifa, Siti Rafa Amirah, Driyanti Rahayu, Sandra Megantara, Muchtaridi Muchtaridi

**Affiliations:** 1 Department of Pharmaceutical Analysis and Medicinal Chemistry, Faculty of Pharmacy, Universitas Padjadjaran, Jl. Raya Bandung-Sumedang Km. 21, Bandung 45363, Indonesia; siti19001@mail.unpad.ac.id (S.R.A.); driyanti.rahayu@unpad.ac.id (D.R.); s.megantara@unpad.ac.id (S.M.); muchtaridi@unpad.ac.id (M.M.); 2 Study Center for Development of Pharmaceutical Preparations, Universitas Padjadjaran, Jl. Raya Bandung-Sumedang Km. 21, Bandung 45363, Indonesia

**Keywords:** statins, HMG-CoA reductase, *Angelica keiskei*, in silico

## Abstract

Statins are cholesterol-lowering drugs with a mechanism of inhibiting 3-hydroxy-3-methylglutaryl-CoA reductase, but long-term use can cause side effects. An example of a plant capable of reducing cholesterol levels is *Angelica keiskei* (ashitaba). Therefore, this study aimed to obtain suitable compounds with inhibitory activity against the HMG-CoA reductase enzyme from ashitaba through in silico tests. The experiment began with screening and pharmacophore modeling, followed by molecular docking on ashitaba’s compounds, statins groups, and the native ligand was (3R,5R)-7-[4-(benzyl carbamoyl)-2-(4-fluorophenyl)-5-(1-methylethyl)-1H-imidazole-1-yl]-3,5-dihydroxyheptanoic acid (4HI). Based on the results of the molecular docking simulations, 15 hit compounds had a small binding energy (ΔG). Pitavastatin, as the comparator drug (ΔG = −8.24 kcal/mol; Ki = 2.11 µM), had a lower ΔG and inhibition constant (Ki) than the native ligand 4HI (ΔG = −7.84 kcal/mol; Ki = 7.96µM). From ashitaba’s compounds, it was found that 4′-O-geranylnaringenin, luteolin, isobavachalcone, dorsmannin A, and 3′-carboxymethyl-4,2′-dihydroxy-4′-methoxychalcone have low ΔG of below −6 kcal/mol. The lowest ΔG value was found in 3′-carboxymethyl-4,2′-dihydroxy-4′-methoxy chalcone with a ΔG of −6.67 kcal/mol and Ki value of 16.66 µM, which was lower than the ΔG value of the other comparator drugs, atorvastatin (ΔG = −5.49 kcal/mol; Ki = 1148.17 µM) and simvastatin (ΔG = −6.50 kcal/mol; Ki = 22.34 µM). This compound also binds to the important amino acid residues, including ASN755D, ASP690C, GLU559D, LYS735D, LYS691C, and SER684C, through hydrogen bonds. Based on the results, the compound effectively binds to six important amino acids with good binding affinity and only requires a small concentration to reduce half of the enzyme activity.

## 1. Introduction

Cholesterol is a waxy, fat-like substance found in all cells within the body, specifically the liver [1]. The presence of excess levels in the bloodstream is a major contributor to plaque formation, which can clog the arteries thus causing heart attacks [2]. A crucial enzyme in cholesterol biosynthesis is 3-hydroxy-3-methylglutaryl (HMG) Co-A reductase, which catalyzes the conversion of HMG-CoA to mevalonic acid [3]. Statins are cholesterol-lowering drugs commonly used by the public due to their efficacy as inhibitors of the HMG-CoA reductase enzyme [4]. Statins have proven potential in reducing LDL levels by 20–60% [5,6,7,8]. Statins are divided into two groups, namely lipophilic and hydrophilic statins. Bitzur et al., 2013, proved that lipophilic statins, including simvastatin and atorvastatin, tend to cause myopathy compared to hydrophilic statins; in addition, long-term use potentially causes side effects, including [9], hepatotoxicity, and increased risk of new-onset type 2 diabetes [10]. As stated in the previous studies, statins could increase the risk of developing diabetes mellitus (DM), but the results have not been statistically significant. In contrast, the West of Scotland Coronary Prevention Study (WOSCOPS) trial showed that statins, specifically pravastatin [11] and pitavastatin [12], have the potential to reduce the risk of diabetes.

Empirically, one of the herbal plants used to reduce cholesterol levels is ashitaba [13]. *Angelica keiskei* (Miq.) Koidzumi, commonly known as ashitaba or Japanese celery, originates from Japan but can be cultivated in Indonesia, specifically in the Mojokerto and Lombok regions. Compounds isolated from ashitaba include various types of chalcones [14,15,16,17,18,19,20,21,22,23,24,25], flavonoids [16,24], and coumarins [23,24]. Previous studies have also reported the presence of sesquiterpenes [26], diterpenes [27], triterpenes [27,28], and various other compounds [23,24,27,28]. According to an in vivo study by Zhang et al., 2015, administration of the ashitaba extract (0.01% and 0.1%, *w*/*w*) for 16 weeks, containing 4-hydroxyderricin and xanthoangelol, effectively suppressed weight gain, as well as reduced plasma cholesterol, glucose, and insulin levels. The treatment increased adiponectin levels, while also reducing triglycerides and liver cholesterol levels in C57BL/6 rats [29]. Moreover, dry extracts of the stems and leaves have been used in health and cosmetic products at a safe dose of approximately 300 mg/kg [13].

Along with technological developments, the discovery and development of new drug candidates classified as complex can be relatively shorter with in silico tests [30]. It is a computational method used in drug design that provides convenience by shortening the process of identifying hit compounds, as well as the selection of hit-to-lead compounds, and prevents problems related to side effects from the drugs being developed [31]. Therefore, this study aimed to carry out an in silico test to determine the inhibitory activity of the active compounds in the ashitaba plant compared with the statin groups (approved by FDA) (Supplementary S1) against the HMG-CoA reductase enzyme using pharmacophore modeling methods and docking simulations.

## 2. Results

### 2.1. Visualization Results of the HMG-CoA Reductase Pharmacophore Feature

**Figure 1:**
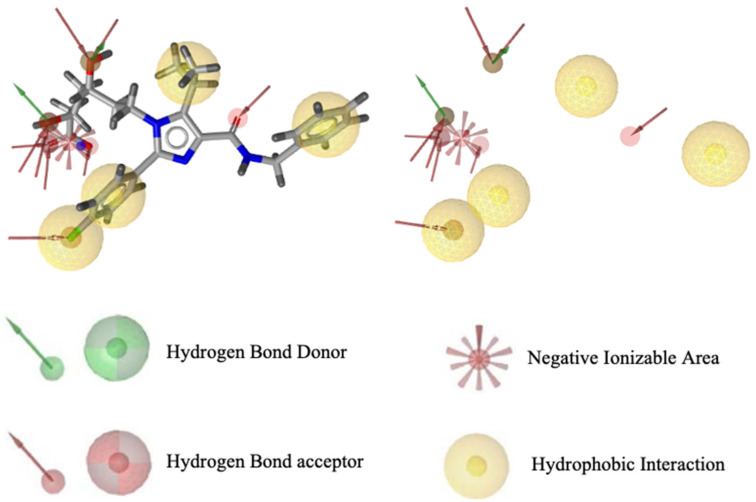
Visualization of pharmacophore features based on HMG-CoA reductase structure.

Pharmacophore modeling was conducted using two methods, namely structure-based drug designing (SBDD) and ligand-based drug designing (LBDD). In the SBDD method, the target protein used as a structural basis was HMG -CoA reductase (PDB ID: 3CCW) complexed with a native ligand (3R,5R)-7-[4-(benzyl carbamoyl)-2-(4-fluorophenyl)-5-(1-methylethyl)-1H-imidazole-1-yl]-3,5-dihydroxy heptanoic acid (C_27_H_31_N_3_O_5_F_1_) or 4HI. This ligand has good pharmacophore features and compatibility with Lipinski’s rules, possessing nine hydrogen bond acceptors, two hydrogen bond donors, four hydrophobic interactions, one negative and zero positive ionized areas (Figure 1), as well as a molecular weight of 496.56 g/mol, and cLogP of 0.80. The results suggest that the pharmacophore features were present in one molecule recognized by the receptor site and are responsible for the biological activity of the compounds.

**Figure 2:**
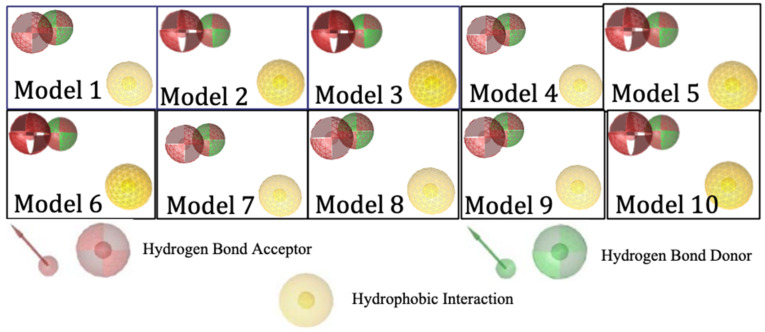
Visualization of ligand-based pharmacophore features.

Ligand-based pharmacophore modeling was carried out using 299 active compounds (actives) HMG-CoA reductase inhibitors and 8884 inactive compounds (decoys) downloaded from the DUD-E (A Database of Useful Decoys). From the results, 10 models were obtained with the pharmacophore features including 2 hydrogen bond acceptors, 1 hydrogen bond donor, and 1 hydrophobic interaction. Figure 2 shows the visualization of the essential features of a ligand-based pharmacophore with biological effects on the target.

### 2.2. The Pharmacophore Model Validation Results

The pharmacophore model needs to be validated before being used as a reference in virtual screening. This step aims to ensure the ability of the pharmacophore model to distinguish between active and inactive compounds. Pharmacophore validation was carried out using 1 structure-based and 10 ligand-based pharmacophore models.

**Figure 3:**
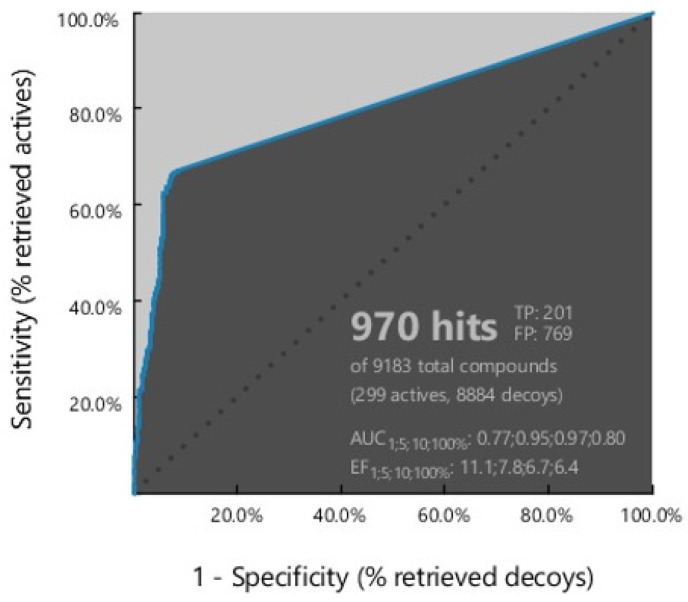
ROC validation curve.

Figure 3 shows the ROC curve from the result of a plot between true positive (sensitivity) on the *Y*-axis and false positive (1-specificity) on the *X*-axis in the best model, namely ligand-based model 7. The area under the ROC curve (Figure 3) was in the range of zero to one or 0–100%. The higher the area under the curve, the better the prediction of the model for active compounds than the decoy [32].

Model 7 recognized 970 hits with an AUC value of 0.8%, an EF value of 6.4, and a GH score of 0.3. The best model selected had a partial area under the curve (pAUC) value of 0.77, 0.95, and 0.97 at 1%, 5%, and 10%, respectively. In addition, this model was sensitive, identifying 201 active compounds out of 209, representing 67% of the total active compounds. The high selectivity and sensitivity indicate that the pharmacophore model is an excellent filter for recognizing HMG-CoA reductase inhibitors. All model validation parameters shown in Table 1 suggest the good quality of the pharmacophore model for the screening stage of hit compounds.

### 2.3. Hit Compound Screening Results

Table 2 showed that 15 out of 36 hit compounds had the highest pharmacophore fit scores. The screening was carried out using a database of tests and standards with a validated pharmacophore model through Ligandscout. These 15 hit compounds were then molecularly docked to HMG-CoA reductase (PDB:3CCW) using the AutoDockTools-1.5.6 software.

### 2.4. The Results of Molecular Docking Validation

**Figure 4:**
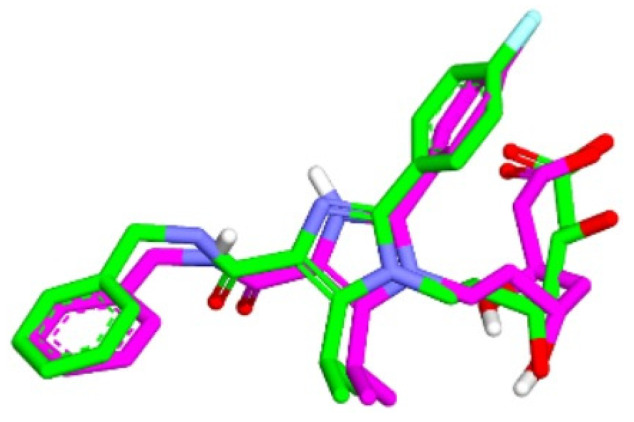
Overlaying of extracted 4HI molecules from HMG-CoA reductase (Green) with re-docking (Pink) (RMSD = 0.98 Å).

The validation of the molecular docking or redocking was carried out between the prepared native ligand and the target protein, namely 3CCW, to identify and ascertain the location of the binding site on the enzyme. The size of the grid box used was 40 × 40 × 40 Å to ensure sufficient space to search for the best position of the ligand on the binding site. An overly large grid box size can lead to non-specific ligand binding and biased test results. The coordinates used were x (3.788), y (30.858), and z (5.445), particularly for positioning the ligand in the active pocket of the HMG-CoA reductase receptor. From this process, an RMSD value of 0.98 Å was obtained, indicating that the molecular docking method met the qualifications and showed good quality of bond pose reproduction because the RMSD value was less than 2.00 Å (Figure 4) [33,34]. The native ligand 4HI binds to the important amino acids ARG590C, ASN755D, ASP690C, and SER565D. It also binds to other amino acids including ALA856D, ASN658C, CYS561D, GLY560D, HIS752D, and LEU853D. In the native ligand, the result of re-docking showed an ΔG of −7.84 with a Ki value of 7.96 µM.

### 2.5. The Molecular Docking Result of Hit Compounds to HMG-CoA Reductase

Molecular docking was carried out to determine the possible interactions between the hit test compounds and important amino acid residues of the target enzyme HMG-CoA reductase in the body compared to native ligands and marketed comparators. The amino acid residues crucial to the catalytic site of the HMG-CoA reductase enzyme according to Sarver et al., 2008, include GLU559, SER565, ARG590, SER684, ASP690, LYS691, LYS735, and ASN755 through hydrogen bonding [7]. This was also supported by Istvan et al., 2001, stating that important amino acids were in the cis loop area, including GLU559, ARG590, SER684, ASP690, LYS691, LYS692, LYS735, and ASN755 [35]. The molecular docking used the grid box arrangement obtained in the validation process to center the test ligands with the active sites on the native ligands. Among the 100× dockings performed, the conformation of the best cluster and the lowest bond energy was selected.

The lowest bond affinity (ΔG) value was found in 3′-carboxymethyl-4,2′-dihydroxy-4′-methoxy chalcone, which is lower than the ΔG value of the comparator drugs atorvastatin and simvastatin. This compound also binds to the important amino acid residues including ASN755D, ASP690C, GLU559D, LYS735D, LYS691C, and SER684C through hydrogen bonds. Pitavastatin, as the comparator drug, had a lower ΔG and inhibition constant than the native ligand.

## 3. Discussion

The process of cholesterol synthesis begins with the condensation of two molecules of acetyl-coenzyme A (acetyl-CoA) to form the intermediate acetoacetyl-CoA, catalyzed by acetyl-CoA acetyltransferase (thiolase enzyme). Furthermore, the reaction of two acetoacetyl-CoA molecules allows the formation of 3 hydroxy-3-methylglutaryl CoA (HMG-CoA) catalyzed by HMG-CoA synthase, then the reduction to mevalonate by the enzyme HMG-CoA reductase (two molecules of NADPH serve as cofactors). These reactions are the rate-limiting steps of the body’s overall cholesterol synthesis and are known as regulatory enzymes [6,36]. Istvan and colleagues successfully performed crystallographic analysis on the catalytic site of human HMGR, a tetramer form, which is composed of two dimers having two active sites (first dimer: monomer 1α and 1β; second dimer: monomer 2α and 2β). They also revealed that the catalytic monomer of hHMGR consists of three domains as follows: N-domain (in N-terminal; residues 460–525; helices Lα1-5), a large L-domain (residues 528–590 and 694–872; helices Lα1-11 and Lβ1-6) and a small S-domain (residues 592–682; helices Sα1-3 and Sβ1-4). The S and L domains are connected by strands, Lβ3 and Sβ1, and a loop (residues 682–694; known as a ‘cis-loop’) that is important in the HMG binding site [36]. On the other hand, Costa and colleagues studied the conformational changes of HMGR in complex with HMG-CoA (binds Lα1-domain; amino acid 528–590) and NADPH (binds S-domains) by constructing a model of human HMGR (hHMGR), which comprised ligand-free hHMGR (Apo form), hHMGR complexed with HMG-CoA and NADPH (holo form), and phosporylated hHMGR. Their study also found that the L2-domain located with the HMG-CoA binding region on the B chain does not interact with substrates and cofactors [37].

Statins are drugs that can bind well to the active side of the HMG-CoA reductase enzyme to inhibit the interaction of the enzyme with the substrate. There is some disagreement regarding the use of statins as anti-cholesterol drugs to increase the occurrence of diabetes. Atorvastatin and simvastatin have an increased risk of DM compared to pravastatin [38]. Still, Cho et al., 2015 [11], reported that there was no significant difference in the risk of T2DM between hydrophilic (pravastatin and rosuvastatin) and lipophilic (simvastatin, atorvastatin, and pitavastatin) statins. The structure and bioavailability of statins do not significantly impact the diabetogenic effect, which is a condition of increased glucose production through carbohydrate/glucose metabolism [11]. Sarver et al., 2008, succeeded in synthesizing imidazole compound 1 (4HI), which has good in vivo efficacy as an inhibitor with an IC50 of 7.9 nM and excellent hepatoselectivity (>1000-fold). This compound, which is complex with HMG-CoA reductase, was crystalized with X-ray diffraction and deposited in rcsb.org (accessed on 3 February 2023). (PDB ID: 3CCW) and used in this study [7].

In this study, we used a pharmacophore modeling approach with the two models namely, the SBDD and LBDD methods [6]. In the SBDD method, the target protein used as a structural basis was HMG-CoA reductase (PDB ID: 3CCW). This ligand has good pharmacophore features and compatibility with Lipinski’s rules, possessing nine hydrogen bond acceptors, two hydrogen bond donors, four hydrophobic interactions, one negative and zero positive ionized areas, as well as a molecular weight of 496.56 g/mol, and cLogP of 0.80. The results suggest that the pharmacophore features were present in one molecule recognized on a receptor site and responsible for the biological activity of compounds.

In the discovery of new compounds, it is necessary to use active compounds and decoy compounds in a ratio of 1:10 [39,40]. Ligand-based pharmacophore modeling was conducted using 299 active compounds (actives) of HMG-CoA reductase inhibitors and 8884 inactive compounds (decoys) downloaded from the DUD-E database (A Database of Useful Decoys) [41]. The DUD-E site already stores data on active compounds which are compounds that are proven to have activity against HMG-CoA reductase enzymes, so they can be used as positive controls. Conversely, decoy compounds are compounds in nature that do not have biological activity against the HMG-CoA reductase enzyme, so they are used as negative controls [40,42]. Pharmacophore validation was carried out using 1 structure-based and 10 ligand-based pharmacophore models. The higher the area under the curve, the better the prediction of the model for active compounds than decoy [32]. The best model, namely ligand-based model 7, was sensitive in identifying 201 active compounds out of 209, representing 67% of the total active compounds. The high selectivity and sensitivity indicate that the pharmacophore model is an excellent filter for recognizing HMG-CoA reductase inhibitors.

All statins are good competitor inhibitors of HMG-CoA reductase because they have pharmacophore groups that show great similarity to the HMG-CoA molecule [43]. Markowska et al., 2020, suggested that statins that have a hydroxy acid form in the side group (pravastatin, atorvastatin, cerivastatin, fluvastatin, pitavastatin, and rosuvastatin) are predicted to have pharmacological activity, while the lactone form in the side group of statins (mevastatin, lovastatin, and simvastatin) could reduce pharmacological activity [8]. Different results appear in this study, based on pharmacophore study, and showed that 36 hit compounds (9 statins and 27 ashitaba’s compounds) had pharmacophore fit scores. This shows that ligand-based model 7 could recognize the active compound (all statins) on the HMG-CoA reductase receptor. We selected 15 compounds (5 statins and 10 ashitaba’s compounds; >46% pharmacophore-fit scores) for molecular docking (Table 2). Pitavastatin and atorvastatin, which have a hydroxy acid form, are hit compounds (fit-pharmacophore results of 48.14% and 48.07%, respectively); however, compounds which have lactone sides such as lovastatin, simvastatin, and mevastatin (compactin) are included in the hit compounds as well (fit-pharmacophore results of 47.55%; 47.44%, and 47.39%), based on pharmacophore screening results.

The molecular docking parameter in this study is the affinity energy/binding energy (ΔG)). Binding energy shows the energy required to bind two molecules; the smaller the energy required to bind to the target protein, the more effective it is. The second parameter is the Ki value. This is a semi-empirical free energy constant usually indicates the inhibition of receptors in micromolar units (µM), which virtually explores the dose concentration. The calculation of the inhibition constant (Ki) value is obtained from the binding energy (ΔG) using the following formula: Ki = exp (ΔG/RT), where R is the universal gas constant (1.985 × 10^−3^ kcal mol^−1^ K^−1^) and T is the temperature (298.15 K). Thereafter, we analyzed the interaction between ligands and receptors at coordinates x = 3.788; y = 30.858; and z = 5.445. The expected interaction is the ligand with key amino acids on the receptor that plays a role in providing biological activity. Amino acid residues which play a role in the catalytic site of the HMG-CoA reductase enzyme, according to Sarver et al., 2008, include GLU559, SER565, ARG590, SER684, ASP690, LYS691, LYS735, and ASN755 through hydrogen bonding [7]. This was also supported by Istvan et al., 2001, who has crystalized six statins with X-ray diffraction and studied the mechanism. They found that when HMG-CoA or CoA substrates are bound to amino acid residues, the C-terminal extension is partially fixed. Still, when the NADP^+,^ HMG, and CoA or NADP^+^ and HMG-CoA substrates are complexed together, the helix at the C-terminal of Lα11 (residues 870 and871) binds to the protein core (known as the cis loop) [36,44]. This configuration would cause the active site to be closed by the Lα11 helix, and no statins would be able to occupy the NADP(H) binding site, which is a finding that is in line with their kinetic studies, as statins are competitive inhibitors of HMG-CoA substrates but are not competitive with NADPH [35,36]. The important amino acids were in the cis loop area, including GLU559, ARG590, SER684, ASP690, LYS691, LYS692, LYS735, and ASN755 (with polar interaction, hydrogen bonding, forming a salt bridge and hydrophobic interaction) [35]. Istvan et al., 2001, also stated that hydrophobic statin compounds with a large molecular weight can occupy the surface of the HMG-CoA binding pocket, causing the substrate of HMG-CoA to HMGR to be blocked [35]. Van der Waals bonds are also formed between the hydrophobic side chains, namely the amino acids LEU562, VAL683, LEU853, ALA856, and LEU857 with statins and ashitaba’s compounds [35].

**Figure 5:**
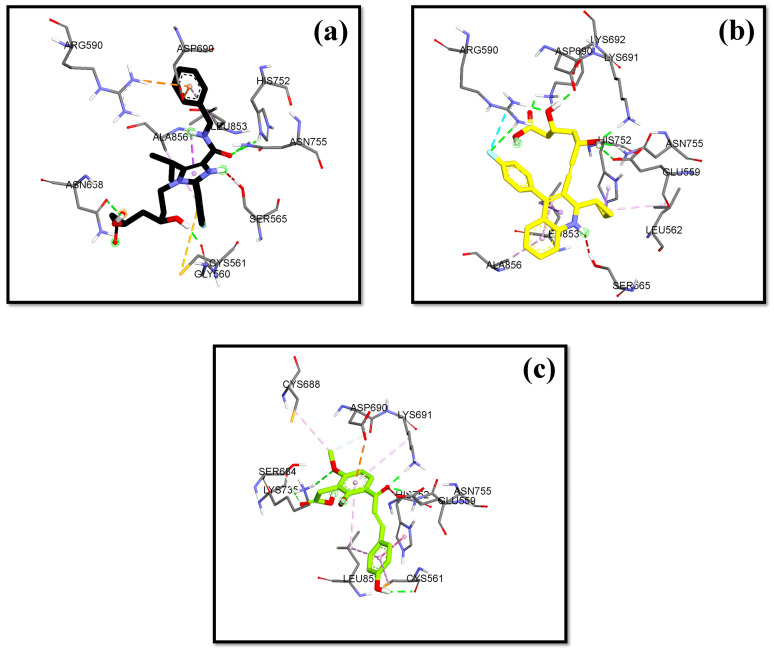
HMG Co-A reductase binding mode with (**a**) 4HI (**b**) Pitavastatin (**c**) 3′-carboxymethyl-4,2′-dihydroxy-4′-methoxy chalcone.

In this research, the native ligand 4HI binds to the important amino acids ARG590C, ASN755D, ASP690C, and SER565D. It also binds to other amino acids including ALA856D, ASN658C, CYS561D, GLY560D, HIS752D, and LEU853D. For the native ligand (4HI), the result of re-docking showed an ΔG of −7.84 with a Ki value of 7.96 µM. Based on the results of molecular docking simulations, 15 hit compounds had a small binding energy (ΔG). Pitavastatin, as the comparator drug, (ΔG = −8.24 kcal/mol; Ki = 2.11 µM) had lower ΔG and inhibition constant (Ki) than the native ligand 4HI. From ashitaba’s compounds, we found that the compounds 4′-O-Geranylnaringenin, luteolin, isobavachalcone, dorsmannin A, and 3′-Carboxymethyl-4,2′-dihydroxy-4′-methoxy chalcone have a low ΔG below −6 kcal/mol (Table 3). The lowest ΔG value was found in 3′-carboxymethyl-4,2′-dihydroxy-4′-methoxy chalcone with a ΔG of −6.67 kcal/mol and Ki value of 16.66 µM, which was lower than the ΔG value of the comparator drugs atorvastatin (ΔG = −5.49 kcal/mol; Ki = 1148.17 µM) and simvastatin (ΔG = −6.50 kcal/mol; Ki = 22.34 µM). This compound also binds to the important amino acid residues including ASN755D, ASP690C, GLU559D, LYS735D, LYS691C, and SER684C through hydrogen bonds (Figure 5). Based on the results, the compound effectively binds to six important amino acids with good binding affinity and only requires a small concentration to reduce half of the enzyme activity.

The C-terminal region (residue 870–886), Flap domain, was involved in the open-closed movement of the active site [37]. In humans, HMGR activity can be modulated by phosphorylation. If phosphorylation occurs at residue 872, close to the important catalytic residue 866, it can reduce protein activity. Istvan’s finding that the position of residue 872 is around the α-phosphate of NADP and the side chain of residue 871, not close to residue 866. Phosphorylation can cause a decrease in affinity for NADPH [36]. Our docking results showed that neither the statins nor/or ashitabas’s compounds bind to residues 870–872 at the C-terminus, so neither statins nor ashitaba’s compounds may be competitive with NADPH.

In this study, we conducted the predictions of physicochemical properties for 15 hit compounds by screening Lipinski’s rule of five (RO5). The RO5 bases pharmacokinetic drug properties such as absorption, distribution, metabolism, and excretion on certain physicochemical properties [45]. A compound can be used as a drug compound if it has a molecular mass of less than 500 Daltons because the molecular weight parameter is related to the polymer chemical properties of the compound. A high polymer molecular weight indicates stronger polymer chemical properties. In addition, it is also related to the compound distribution process, because compounds with molecular weights of more than 500 Da cannot passively diffuse so in penetrating biological membranes, the absorption of compounds in the body becomes longer [46,47]. Second, the partition coefficient (logP) should be less than five. A large logP value indicates that the compound is hydrophobic and tends to have a high level of toxicity, retained longer in the lipid bilayer, and distributed more widely in the body so that the compound’s affinity to the enzyme is reduced. A negative logP value would make it difficult for the compound to pass through the lipid bilayer membrane [48], which allows for rapid interaction of the molecule with the water solvent [47]. Finally, the number of hydrogen bond donors should be less than 5, and hydrogen bond acceptors should be less than 10. This was because the higher the hydrogen bond capacity, the higher the energy required in the absorption process, so that the ability of the hydrogen bond acceptor is reduced [48]. The physicochemical profiles of compounds would be similar across industries if the testing methodology, selection criteria, and compounds screened were similar. The results of Lipinski’s rule of five analysis on 15 compounds (Supplementary S2) that fit the pharmacophore modeling could provide data on the physicochemical properties of these compounds, which may be useful in subsequent in vitro or in vivo testing. We conduct this RO5 as a tool to guide early-stage drug discovery.

## 4. Materials and Methods

### 4.1. Instruments

The tools used in computational testing include hardware and software with different functions and purposes. The hardware comprised a personal laptop with AMD A9-9420e Radeon R5 processor specifications, 5 computer cores 2C + 3G 1.80 GHz; 8.00GB RAM; system type 64 bit, x64-based processor; and Windows 10 Pro version 22H2 operating system. On the other hand, software was obtained free of charge for academic users. These included the following: LigandScout 4.4.5 (InteLigand, https://ligandscout.software.informer.com/, Vienna, Austria; accessed on February–May 2023) for pharmacophore modeling and screening. AutoDockTools-1.5.6 (The Scripps Research Institute, http://autodock.scripps.edu/), Command Prompt, and Notepad-11.2307.27.0 were used for ligand and receptor preparation, validation, as well as molecular docking simulations. The BIOVIA Discovery Studios 2021 Client (Dassault Systems Biovia, https://discover.3ds.com/discovery-studio-visualizer-download) was used for the preparation of receptors with native ligands, to visualize the results of complex molecular docking, bonding between ligands and receptors, geometrical optimization, and overlays in the validation process. Moreover, ChemDraw Ultra 12.0 (PerkinElmer Inc., http://www.cambridgesoft.com/) was used to draw the 2D structures of the ligand compounds. Chem3D Pro 12.0 (PerkinElmer Inc., http://www.cambridgesoft.com/) was used to convert the 2D structure ligand into a 3D shape and optimize the energy. Open Babel-2.4.1 (Open Babel community http://openbabel.org/docs/Installation/install.html) was used to convert chemical file formats, while the DUD-E site accessed on https://dude.docking.org/ was used to get active and decoy databases. The NCBI site, accessed at https://www.ncbi.nlm.nih.gov/, was used to search for recorded test compounds. The Protein Data Bank site accessed at https://www.rcsb.org/structure/3CCW was used to search for the target receptor code and SwissADME at http://www.swissadme.ch/index.php was utilized for the prediction of physicochemical properties.

### 4.2. Materials

The materials used in this study included the three-dimensional structure of the target receptor resulting from an X-ray crystallographic depiction of the HMG-CoA reductase enzyme in the Homo sapiens/human organism (PDB ID: 3CCW), with a good resolution of 2.10 Å downloaded through the Protein Data Bank (http://www.rcsb.org/, accessed on January–May 2023). Molecular docking validation was carried out using the BIOVIA Discovery Studios 2021 Client. The ligand used as a positive control/reference drug was the three-dimensional structure of (3R,5R)-7-[4-(benzyl carbamoyl)-2-(4-fluorophenyl)-5-(1-methylethyl)-1H-imidazole-1-yl]-3,5-dihydroxyheptanoic acid (4HI), which was separated from the target protein using the BIOVIA Discovery Studios 2021 Client program, as well as the statins (Supplementary S1) available on the market. In addition, the tested ligands used were two-dimensional structures of 115 secondary metabolites isolated from ashitaba plant, namely 42 chalcones, 7 flavanones, 3 flavones, 5 flavonols, 39 coumarins, 2 phenolics, 3 sesquiterpenes, 1 diterpene, 3 triterpenes, and 12 other compounds obtained by preparation using the ChemDraw Ultra 12.0 and Chem3D Pro 12.0.

### 4.3. Methods

This study was carried out using the in silico (computation-based) method, with initial stages including modeling, validation, and pharmacophore screening using LigandScout 4.4.5. Other steps were molecular docking simulations, prediction of pharmacokinetic properties and ligand toxicity, as well as reviewing the achievement of Lipinski’s Rule of Five (RO5).

### 4.4. Pharmacophore Modeling

Pharmacophore modeling was carried out through the Structure-Based Drug Design (SBDD) and Ligand-Based Drug Design (LBDD) methods. Structure-based pharmacophore modeling was conducted by opening and then resetting LigandScout 4.4.5 first to the default settings. In the ‘Structure-Based’ section, the 3D structure of the HMG-CoA reductase target protein with the 3CCW PDB code was downloaded. Following this, the ‘Create Pharmacophore’ icon was clicked until the pharmacophore features of the native ligands were visible to the target protein.

In the ligand-based pharmacophore modeling, the material needed during the prepared test included three databases, namely active, decoy, and test compounds. The database was made by opening the LigandScout 4.4.5 program and clicking the ‘Ligand Based’ column. Active/decoy files obtained from the DUD-E website were opened for the active/decoy database, while for the test database, the test compound was opened to be tested and optimized in 3D form. In the ‘Type’ section, all compounds were in the ‘training’ form for active and decoy databases, while for the database, all compounds were in the ‘Test’ form. The files were stored in the LDB format, and the pharmacophore modeling stage was continued after all databases were deemed ready.

In the ‘Ligand Based’ section of the active database previously opened, the cluster was made on all compounds, then the sequence of compounds was ‘sorted by cluster’. This was achieved by clicking the ‘Cluster ID’ column, and one type of ‘training’ file was selected from each cluster. The ‘Create Pharmacophore’ icon was clicked, and 10 pharmacophore models were stored in the PMZ format.

### 4.5. Pharmacophore Validation

The pharmacophore model previously obtained was tested for validity by moving to the ‘Screening’ column by clicking the ‘Copy to Other Perspective’, and then the ‘Screening Perspective’ icon. In the ‘Screening’ column, ‘Load Screening Database’ was clicked to select the active and decoy database with the LDB format previously created. The active database entered was marked in green, while the decoy was marked in red. The ‘Perform Screening’ icon was clicked to allow LigandScout 4.4.5 to perform the screening process. To visualize the validity of the ‘Plot ROC Curve’, the AUC value was observed, and then the best ROC and EF from the 10 models were selected.

### 4.6. Screening of Hit Compounds

In the ‘Screening’ column, ‘Load Screening Database’ was clicked to select the test database of the test compound previously created. The database is marked in green and in the ‘Ligand Based’ section, the best model was moved to the ‘Screening’ column. The ‘Perform Screening’ icon was then clicked to derive a hit compound.

### 4.7. Molecular Docking Simulation

#### 4.7.1. Separation of Native Ligands and Receptors

The HMG-CoA Reductase enzyme as macromolecules (receptors) originating from humans, with the PDB code 3CCW, was downloaded on the bank data protein website (https://www.rcsb.org/, accessed on January–May 2023). The receptors were cleaned from other components not needed in docking protocols, including water molecules and ligands using BIOVIA Discovery Study 2021 Client (https://discover.3ds.com/discovery-studio-visualizer-download, accessed on May–July 2023) and saved to the PDB format. This step was also carried out to separate the native ligands from the macromolecules.

#### 4.7.2. Ligand Preparation

The ligand structure was designed by opening the ChemDraw Ultra 12.0 to form 113 structures of the compound from ashitaba and on ChemACX.Com, accessed on January–May 2023). Structural modification was carried out by adding or removing according to the structure source of compounds on the NCBI site or the reference literature, and the file was then stored in a CDX format. Furthermore, 2D structures/images were converted to 3D, followed by the minimization of energy (MM2) using the Chem3D Pro 12.0 to obtain a more stable structure. The files were stored in the PDB format. Autodock Tools-1.5.5 was used to edit the PDB format ligand by adding hydrogen atoms then selecting ‘Merge Non-Polar’, computing Gasteiger charge, and adding torsion. The files were stored in the PDBQT format.

#### 4.7.3. Macromolecule Preparation

Macromolecule preparation was performed using Autodock Tools-1.5.5, followed by editing the receptor file in a PDB format by adding hydrogen and then selecting ‘Polar Only’ and Kollman charge. The files were stored in the PDBQT format. To determine the binding site ligand in the receptor, the grid parameter was determined using Autodock Tools-1.5.6. The receptors were selected as macromolecules, and the ‘map type’ was set by selecting ligands. The search for active site receptors was carried out by clicking ‘Center on Ligand’ to obtain the size of the Center Grid Box (X, Y, and Z). This size must be used in adjusting between the active side of the receptor and other ligands. The files were stored in the GPF (Grid Parameter File) format.

#### 4.7.4. Molecular Docking

PDBQT format receptors were first designated as macromolecules, then as ligands for the docking process. Docking was performed using the Lamarckian GA (Genetic Algorithm) with parameters set for 100 runs, followed by adjustment to the default docking parameter, and files were then stored in the DPF (Docking Parameter File) format. Furthermore, the molecular docking process was carried out using the Command Prompt feature. Determination of the ligand conformation with the results of the best molecular docking was conducted by selecting ligand conformation with the lowest bond energy level. Free binding energy values and RMSD were derived from the histogram in the DLG format docking file using Notepad. The conformations were then made into a complex file in PDB format. Complex files were visualized using the BIOVIA Discovery Studio 2021 Client to observe the overlay display of re-docking results and ligand interactions with receptors in 2D or 3D diagrams. Docking visualization is needed to find out the locations of the ligand and the receptor. In addition to visualizing the inhibited side, visualization also helps avoid errors in the docking process.

#### 4.7.5. Overview of Lipinski’s Rule of Five (RO5)

Lipinski’s Rule of Five (RO5) was conducted to guide early-stage drug discovery. Predictions of the physicochemical properties were conducted online on the SwissADME website (http://www.swissadme.ch/index.php, accessed on January–May 2023). The candidates for active compounds in the drugs should comply with Lipinski’s five rules, namely molecular weight ≤ 500, hydrogen bonds acceptor ≤ 10, hydrogen bond donors ≤ 5, and logP value ≤ 5 (or MlogP ≤ 4.15) [49].

## 5. Conclusions

In conclusion, for the native ligand (4HI), the result of re-docking showed an ΔG of −7.84 with a Ki value of 7.96 µM. Based on the results of molecular docking simulations, 15 hit compounds had a small binding energy (ΔG). Pitavastatin, as the comparator drug, had lower ΔG and Ki values than the native ligand 4HI. The best docking result of ashitaba’s compound, 3′-carboxymethyl-4,2′-dihydroxy-4′-methoxy chalcone has one hydrophobic interaction, one hydrogen bond donor, and two hydrogen bond acceptors based on modeling with a pharmacophore fit score of 46.90%. This compound also had six hydrogen interactions with the important amino acid residues, ASN755D, ASP690C, GLU559D, LYS735D, LYS691C, and SER684C, with a ΔG value of −6.67 kcal/mol and a Ki value of 16.66 µM. Our docking results showed that neither statins nor/or ashitabas’s compounds bind to residues 870–872 at the C-terminus, so neither statins nor ashitaba’s compounds may be competitive with NADPH. The results of Lipinski’s rule of five analysis on 15 compounds provided data on the physicochemical properties of these compounds, which may be useful in subsequent in vitro or in vivo testing. We used this RO5 as a tool to guide early-stage drug discovery.

## Figures and Tables

**Table 1:** Pharmacophore Modeling Validation Parameters.

Parameter	Result
Total compounds in database (D)	9183
Active total in database (A)	299
Total hits (Ht)	970
Active hits (Ha)/True Positive (TP)	201
False Positives (FP)	769
True Negatives (TN = D − FP)	8414
False Negatives (FN = A − Ha)	98
Area Under ROC Curve (AUC)	0.8
Enrichment Factor (EF = [Ha × D]/[Ht × A])	6.4
Goodness of Hit score $\left(\mathrm{GH}=[\frac{\left\{\mathrm{Ha}\left(3A+\mathrm{Ht}\right)\right\}}{4\mathrm{Ht}A}]\{1-\frac{\left(\mathrm{Ht}-\mathrm{Ha}\right)}{\left(D-A\right)}\}\right)$	0.3
Sensitivity (TPR = TP/A)	0.67
Specificity (TNR = TN/D)	0.92
Accuracy (ACC = (TP + TN)/(A + D))	0.91

**Table 2:** Pharmacophore Screening Result.

No.	Compound	Fit-Pharmacophore Score (%)
1	4HI	47.05
2	Pitavastatin	48.14
3	Atorvastatin	48.07
4	Lovastatin	47.55
5	Simvastatin	47.44
6	Mevastatin (Compactin)	47.39
7	4′-*O*-Geranylnaringenin	47.98
8	Luteolin	47.70
9	Cynaroside	47.47
10	7-*O*-Methyl prostratol F	47.40
11	Xanthokeismin A	47.31
12	Daucosterol	46.92
13	Isobavachalcone	46.92
14	Dorsmannin A	46.91
15	3′-Carboxymethyl-4,2′-dihydroxy-4′-methoxy chalcone	46.90
16	Xanthokeistal A	46.71

**Table 3:** Molecular docking results of the hit compounds.

No.	Compound	ΔG (kcal/mol)	Ki (µM)	Important Amino Acid Residues [7]	Other Amino Acid Residues
**Reference Drugs**					
1	Pitavastatin	−8.24	2.11	ARG590C, ASN755D, ASP690C, GLU559D, LYS691D, SER565D	LEU562D, LYS692C, HIS752D, LEU853D, ALA856D
2	Atorvastatin	−5.49	1148.17	ARG590C, GLU559D, LYS735D, SER684C	CYS561D, ALA564D, LEU853D
3	Lovastatin	−6.88	10.65	ARG590C, ASP690C, LYS691C	LEU562D, HIS752D, LEU853D, ALA856D
4	Simvastatin	−6.50	22.34	ARG590C, ASP690C, LYS735D, SER684C	SER661C, VAL683C, LYS692C, HIS752D, LEU853D, LEU857D
5	Mevastatin (Compactin)	−6.86	11.82	ARG590C, LYS691C, SER565D	CYS561D, LEU562D, MET657C, LEU853D, LEU857D
**Ashitaba’s Compounds**					
1	4′-*O*-Geranylnaringenin	−6.48	20.24	ARG590C, ASN755D, ASP690C, LYS691C, SER684C	CYS561D, LEU562D, ASN686C, ALA751D, HIS752D, LEU853D, ALA856D
2	Luteolin	−6.03	40.69	ASP690C, GLU559D, LYS735D, SER565D, SER684C	LEU562D, ALA751D, HIS752D, LEU853D
3	Cynaroside	−5.43	153.65	ARG590C, ASP690C, ASN755D, GLU559D	LYS692C, ALA751D, LEU853D, ALA856D
4	7-*O*-Methyl prostratol F	−5.84	70.70	ASN755D, ASP690C, GLU559D, LYS735D, LYS691C, SER684C	CYS561D, CYS688C, HIS752D, LEU853D
5	Xanthokeismin A	−5.11	202.71	ARG590C, ASN755D, ASP690C, LYS691C, SER565D, SER684C	CYS561D, LEU562D, VAL683C, LEU853D, ALA856D, LEU857D
6	Daucosterol	−5.41	203.28	ARG590C, ASN755D, ASP690C, GLU559D, LYS735D, LYS691C	CYS561D, ALA564D, ALA751D, ALA856D, LEU853D
7	Isobavachalcone	−6.00	42.71	ARG590C, ASP690C, GLU559D, LYS691C, SER565D	MET657C, ALA751D, SER852D, LEU853D, ALA856D
8	Dorsmannin A	−6.65	15.78	ARG590C, ASP690C	CYS561D, LEU562D, LEU853D, ALA856D
9	3′-Carboxymethyl-4,2′-dihydroxy-4′-methoxy chalcone	−6.67	16.66	ASN755D, ASP690C, GLU559D, LYS735D, LYS691C. SER684C	CYS561D, CYS688C, HIS752D, LEU853D
10	Xanthokeistal A	−4.77	487.55	ASP690C, LYS691C, SER565D	CYS561D, LYS692C, ALA751D, HIS752D, LEU853D, ALA856D

## Data Availability

The data generated in the present study may be requested from the first author upon reasonable request.

## Supplementary Materials

The following supporting information can be downloaded at: https://www.mdpi.com/article/10.3390/molecules29132983/s1, Supplementary S1. Structure of Statins Group. Supplementary S2. Lipinski Rule of Five (RO5) Hit Compound.
