# Supplementary material for: Pharmacophore Modeling and Binding Affinity of Secondary Metabolites from Angelica keiskei to HMG Co-A Reductase
Source: Molecules. 2024 Jun 23;29(13):2983. doi: 10.3390/molecules29132983 (PMC11243442; doi:10.3390/molecules29132983)
Supplement: Supplementary file 1 [file molecules-29-02983-s001.zip › molecules-3060999-supplementary.pdf]

Supplementary S1. Structure of Statins Group

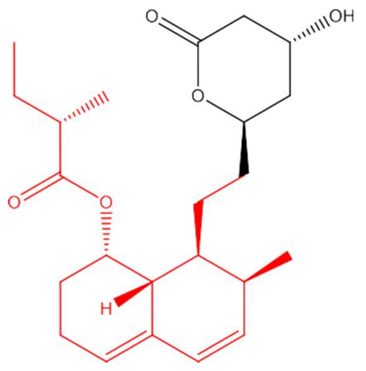

Mevastatin (Compactin)
$C_{23}H_{34}O_5$/MW 390.51

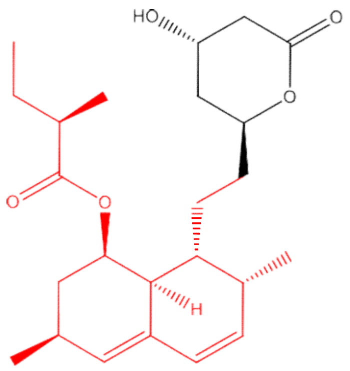

Lovastatin
$C_{24}H_{36}O_5$/MW 404.54

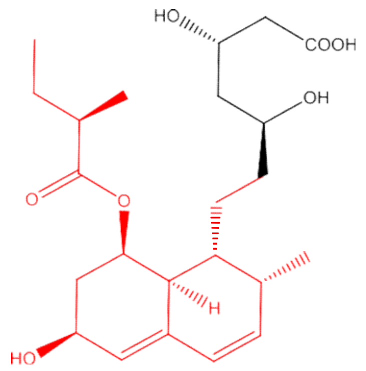

Pravastatin
$C_{23}H_{36}O_7$/MW 424.53

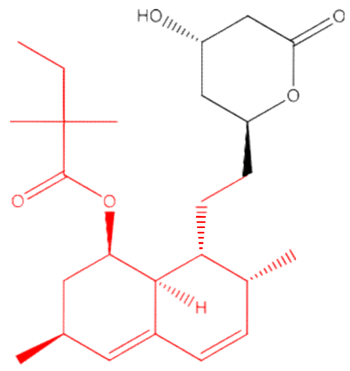

Simvastatin
$C_{25}H_{38}O_5$/MW 418.57

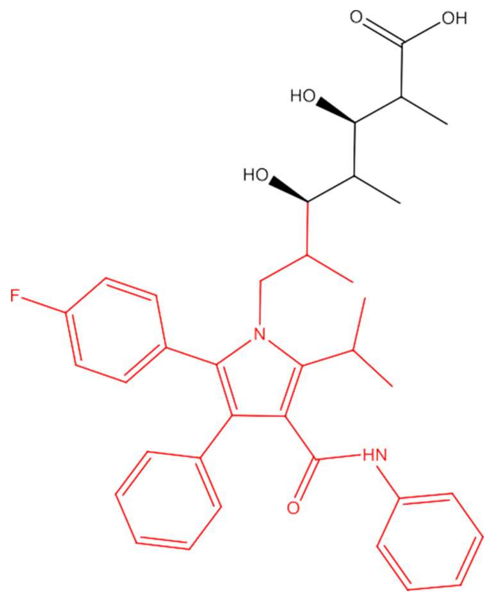

Atorvastatin
$C_{33}H_{35}O_5$/MW 558.64

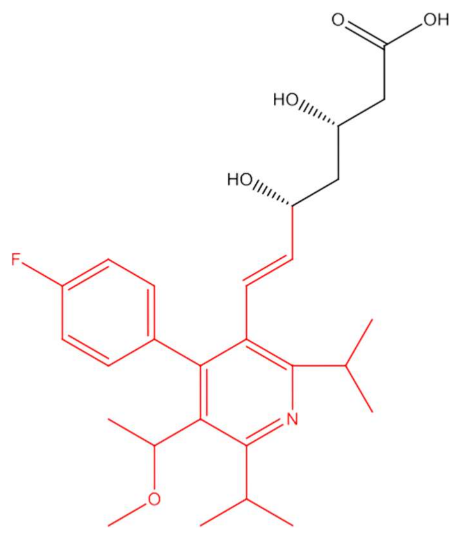

Cerivastatin
$C_{26}H_{34}FNO_5$/MW 459.55

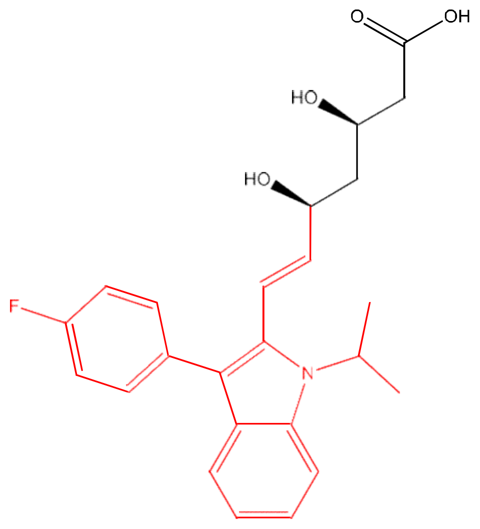

Fluvastatin
$C_{24}H_{26}FNO_4$/MW 411.47

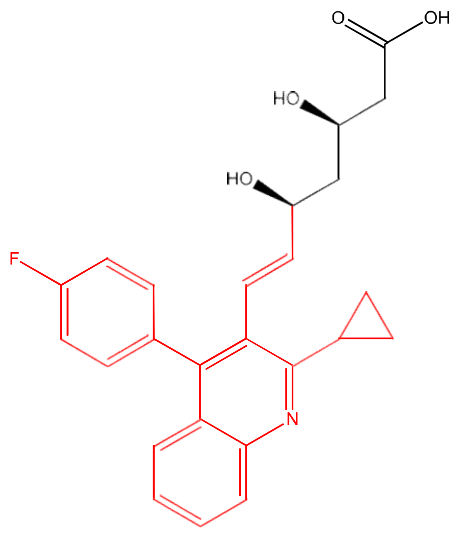

Pitavastatin
$C_{25}H_{24}FNO_4$/MW 421.46

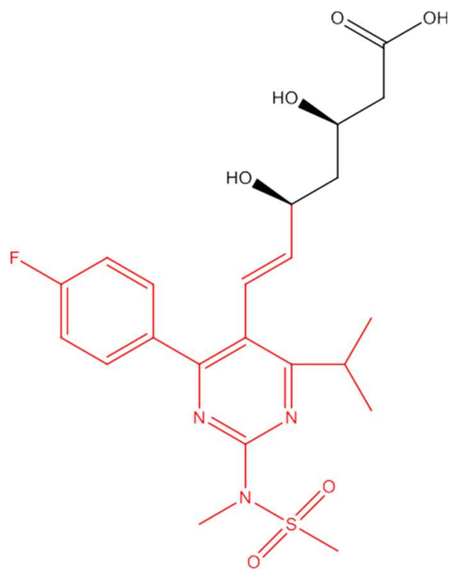

Rosuvastatin
$C_{22}H_{28}FN_3O_6S$/MW 481.54

Supplementary S2. Lipinski Rule of Five (RO5) Hit Compound

No.	Compound Name	Molecular weight (≤ 500 g/mol)	Log P (≤ 4.15)	Hydrogen Bond Donor (NH or OH ≤ 5)	Hydrogen Bond Acceptor (N or O ≤ 10)
**Reference drugs**					
1	4HI	497.56	2.17	4	7
2	Atorvastatin	600.72	4.02	4	6
3	Lovastatin	404.54	3.57	1	5
4	Simvastatin	418.57	3.77	1	5
5	Compactin	390.51	3.36	1	5
6	Pitavastatin	421.46	3.09	3	6
**Ashitaba's compounds**					
1	4′-O-Geranylnaringenin	408.49	2.82	2	5
2	Luteolin	286.24	-0.03	4	6
3	Cynaroside	448.38	-2.1	7	11
4	7-O-Methyl prostratol F	422.51	3.03	2	5
5	Xanthokeismin A	300.39	2.94	2	3
6	Daucosterol	576.85	3.96	4	6
7	Isobavachalcone	324.37	2.7	3	4
8	Dorsmannin A	324.37	2.38	2	4
9	3′-Carboxymethyl-4,2′-dihydroxy-4'-methoxy chalcone	328.32	1.41	3	6
10	Xanthokeistal A	412.48	2.33	3	6
